# Regional block versus general anaesthesia for caesarean section and neonatal outcomes: a population-based study

**DOI:** 10.1186/1741-7015-7-20

**Published:** 2009-04-29

**Authors:** Charles S Algert, Jennifer R Bowen, Warwick B Giles, Greg E Knoblanche, Samantha J Lain, Christine L Roberts

**Affiliations:** 1 Kolling Institute of Medical Research, Clinical and Perinatal Population Health Research, University of Sydney, Australia; 2 Department of Neonatology, Royal North Shore Hospital, Sydney, Australia; 3 Department of Obstetrics and Gynaecology, Royal North Shore Hospital, Sydney, Australia; 4 Department of Anaesthetics, Royal North Shore Hospital, Sydney, Australia

## Abstract

**Background:**

Anaesthesia guidelines recommend regional anaesthesia for most caesarean sections due to the risk of failed intubation and aspiration with general anaesthesia. However, general anaesthesia is considered to be safe for the foetus, based on limited evidence, and is still used for caesarean sections.

**Methods:**

Cohorts of caesarean sections by indication (that is, planned repeat caesarean section, failure to progress, foetal distress) were selected from the period 1998 to 2004 (*N *= 50,806). Deliveries performed under general anaesthesia were compared with those performed under spinal or epidural, for the outcomes of neonatal intubation and 5-minute Apgar (Apgar5) <7.

**Results:**

The risk of adverse outcomes was increased for caesarean sections under general anaesthesia for all three indications and across all levels of hospital. The relative risks were largest for low-risk planned repeat caesarean deliveries: resuscitation with intubation relative risk was 12.8 (95% confidence interval 7.6, 21.7), and Apgar5 <7 relative risk was 13.4 (95% confidence interval 9.2, 19.4). The largest absolute increase in risk was for unplanned caesareans due to foetal distress: there were five extra intubations per 100 deliveries and six extra Apgar5 <7 per 100 deliveries.

**Conclusion:**

The infants most affected by general anaesthesia were those already compromised *in utero*, as evidenced by foetal distress. The increased rate of adverse neonatal outcomes should be weighed up when general anaesthesia is under consideration.

## Background

Internationally, obstetric anaesthesia guidelines recommend spinal and epidural over general anaesthesia (GA) for most caesarean sections (CSs) [1,2]. The primary reason for recommending regional blocks is the risk of failed endotracheal intubation and aspiration of gastric contents in pregnant women who undergo GA [3]. While there is evidence that GA is associated with an increased need for neonatal resuscitation [4], evidence about specific delivery indications and about neonatal outcomes subsequent to resuscitation is limited. Previous studies have usually been single hospital-based and lacked power to confidently detect differences in a rare neonatal outcome such as a low 5-minute Apgar score (Apgar5), particularly among sub-groups such as emergency deliveries. Observational studies, generally unstratified by risk, are subject to confounding since emergencies such as an antepartum haemorrhage can be both an indication for GA and the cause of poor infant status at birth. A *Cochrane Database of Systematic Reviews *of anaesthesia for CS included only two randomised studies with a total of 10 Apgar5 <7 events, and one trial with oxygen therapy as an outcome [5]. That meta-analysis, and another which used cord blood acid-base status as the outcome [6], concluded that there was no evidence that regional anaesthesia was superior to GA in regard to neonatal outcomes.

Although the use of GA for CS has declined while the use of regional techniques has increased [7], both planned and unplanned CS continue to be performed under GA. GA can be thought to be the quickest anaesthesia method in an emergency since it avoids the possibility of a failed regional block. The purpose of this study was to determine the relative risks of neonatal resuscitation with intubation and of an Apgar5 <7 when CS was performed under GA compared with a regional block stratified by specific indications for CS and levels of risk to the foetus. A further purpose was to examine whether the risk of adverse events varied by level of hospital.

## Methods

The study population included all liveborn infants delivered by CS in New South Wales (NSW), Australia from 1 January 1998 to 31 December 2004. Data were obtained from two de-identified linked population databases. The Midwives Data collection (MDC) is a legislated surveillance system of all births in NSW of ≥ 20 weeks gestation or ≥ 400 g birth weight. The Admitted Patient Data Collection has records of all hospital admissions, including ICD10 diagnostic codes related to the admission. Linked MDC and hospital birth admissions were available from 1998 to 2004. Non-linked data on anaesthesia for CS was also available from the MDC for the years 2005 and 2006. The study was approved by the NSW Population and Health Services Research Ethics Committee and the University of Sydney Human Research Ethics Committee.

The MDC collects information on maternal characteristics, pregnancy, labour, delivery and infant outcomes. It includes tick boxes for spinal, epidural and/or GA at delivery. In this study, regional block included any record where spinal and/or epidural anaesthesia (including combined spinal/epidural) was recorded. The outcome and exposure measures in this study are reliably reported on the MDC [8,9]. Compared with medical records, MDC reporting had excellent agreement beyond chance (kappa >0.75) for GA for CS, epidural and spinal anaesthesia, neonatal resuscitation and Apgar5 score, and almost perfect agreement for CS. Only four CS deliveries in the study period did not have type of anaesthesia recorded and only 84 (0.06%) were missing an Apgar5 score.

A CS was categorised as 'planned' if performed prior to the onset of labour and as 'unplanned' if performed after labour had begun. Deliveries where a regional block was recorded in addition to GA are referred to as 'conversions' to GA and presumably represent failed regional blocks. Hospitals were grouped into three categories: 'large public', which are public hospitals providing high-risk obstetric care and 24-hour on-site anaesthetic staff, 'other public' and 'private'.

The primary infant outcomes were resuscitation requiring intubation of the neonate at the time of delivery and the 5-minute Apgar score (Apgar5), dichotomised as <7 or ≥ 7. An Apgar5 score of <7 is associated with increased risk of infant mortality and neurological impairment [10]. The rates of these outcomes for infants exposed to CS under GA were compared with CS under any regional block technique. The GA category in the analyses included those deliveries where both GA and regional block were used (converted regional blocks). To control for confounding by indication, comparisons were made for three pre-specified 'risk' groups, defined by the indications for CS: 'low-risk' pregnancies were planned repeat CS; 'moderate-risk' pregnancies were for failure to progress and where foetal distress was absent; 'high-risk' pregnancies were unplanned CS for foetal distress. All three risk groups were restricted to pregnancies with the following (low foetal risk prior to delivery) characteristics: maternal age 20 to 44 years, gestation 38 to 41 completed weeks, singleton pregnancy. Pregnancies with reported hypertension, oligohydramnios, polyhydramnios, antepartum haemorrhage, or care for a suspected foetal abnormality were excluded as these conditions could have been associated with both anaesthesia choice and neonatal outcome. Births were further restricted to non-breech presenting live births >10th percentile of size for gestational age. Since the 10th percentile for females at 38 weeks in NSW is 2660 g, this was the minimum birth weight for inclusion in this analysis. Relative risks (RRs) and risk differences and their 95% confidence interval (CI) were calculated for each indication and/or risk group. The risk difference is the absolute difference in outcome rates between exposure groups and, in this study, measures the excess rate of adverse outcomes attributable to GA.

To examine the potential impact of variation in level of anaesthetic care available, the risks of intubation and an Apgar5 <7 were further stratified by hospital level for each risk group. The risk differences for each hospital category were calculated and presented as forest plots, and the heterogeneity of effect was assessed using the I-squared statistic (I^2^) [11]. The I^2 ^value estimates the percentage of variation across sub-groups (hospital levels, in this case), which is due to true heterogeneity of effect rather than chance.

## Results

From 1998 to 2004, there were 592,125 deliveries. Annual deliveries declined by 0.9% from 85,072 in 1998 to 84,288 in 2004. The number of women delivered by CS rose steeply, up 41.5%, from 16,216 in 1998 to 22,904 in 2004. Over this period, the percentage of CS performed under GA declined (Table 1). The decrease in use of GA was greater for planned CS than for unplanned CS (25.0% versus 18.3%). Private hospitals had the lowest rate of GA use and other public hospitals had the highest rate. Data from the non-linked 2006 MDC showed a further decline in the use of GA: 1654 unplanned CS under GA (15.3%) and 1627 planned CS under GA (10.5%). The rate of failed regional blocks fell for both planned and unplanned CS, but the absolute numbers increased slightly due to the large overall increase in CS deliveries.

**Table 1:** Caesarean section delivery frequencies for New South Wales 1998 and 2004.

	**1998** ***N *(%)**	**2004** ***N *(%)**	**Change in frequency relative to 1998 (%)**
All planned CS	8800	12,930	+46.9
- intended GA	2193 (24.9)	1597 (12.4)	-27.2
- GA after regional block	153 (1.7)	162 (1.3)	+5.9
All unplanned CS	7416	9974	+34.5
- intended GA	1937 (26.3)	1468 (14.8)	-24.2
- GA after regional block	329 (4.5)	384 (3.9)	+16.7
			
CS in large public hospital	5913	8264	+39.8
- under GA	1397 (23.6)	1404 (17.0)	+0.5
CS in other public hospitals	6025	6954	+15.4
- under GA	2401 (39.9)	1564 (22.5)	-34.9
CS in private hospitals	4278	7686	+79.7
- under GA	814 (19.0)	643 (8.4)	-21.0

CS, caesarean section; GA, general anaesthesia.

Of the 137,987 CS deliveries during the study period, 69,437 were pregnancies without apparent foetal risk factors prior to delivery. From these deliveries, three specific CS indication groups were selected, totalling 50,806 live births. The RR and risk differences for resuscitation requiring intubation and of an Apgar5 <7 for the specific CS indication groups are shown in Table 2. For planned repeat CS deliveries performed under regional block, both intubation (0.09%) and an Apgar5 of <7 (0.17%) were rare events. The RRs when GA was used for repeat CS were greatly increased, and in these otherwise low-risk deliveries, the excess risk attributable to GA was one intubation and two Apgar5 <7 scores per 100 deliveries. The excess risk attributable to GA increased with the urgency of the indication for delivery, so that for foetal distress deliveries there were five extra intubations per 100 deliveries under GA and six extra Apgar5 <7 scores. Among the infants who did require intubation, those that had been delivered with GA had higher rates of an Apgar5 <7 compared with regional block: 42% versus 20% for planned repeat CS (*P *= 0.06), 51% versus 21% for failure to progress (*P *< 0.01), and 57% versus 34% for foetal distress as the indication (*P *< 0.001).

**Table 2:** Effect of anaesthesia type, by caesarean section indication, on relative risk of neonatal outcomes.

**Riskgroup/Indication**	**Outcome**	**General anaesthesia events/*N *(rate %)**	**Regional block events/*N *(rate %)**	**Relative risk if general anaesthesia used (95% confidence interval)**	Risk difference* (%) (95% confidence interval)
**Low risk**: planned repeat	Resuscitation w/intubation	46/4149 (1.11)	20/23,139 (0.09)	12.8 (7.6, 21.7)	1.0 (0.7, 1.3)
caesarean					
section	Apgar5 <7	96/4146 (2.32)	40/23,134 (0.17)	13.4 (9.2, 19.4)	2.1 (1.7, 2.6)
					
**Moderate risk**: unplanned	Resuscitation w/intubation	71/2320 (3.06)	68/13,449 (0.51)	6.1 (4.3, 8.5)	2.6 (1.8, 3.3)
caesarean					
section for failure to progress	Apgar5 <7	95/2319 (4.10)	70/13,446 (0.52)	7.9 (5.8, 10.7)	3.6 (2.8, 4.4)
					
**High risk**: unplanned	Resuscitation w/intubation	139/2058 (6.75)	105/5759 (1.82)	3.7 (2.9, 4.8)	4.9 (3.8, 6.1)
caesarean section for foetal					
distress during labour	Apgar5 <7	158/2054 (7.69)	95/5757 (1.65)	4.7 (3.6, 6.0)	6.0 (4.8, 7.2)

*Rate in general anaesthesia sub-group minus the rate in the regional block sub-group, representing the excess number of adverse outcomes per 100 deliveries under general anaesthesia.

A separate group of 3473 small-for-gestational-age infants delivered at 38 to 41 weeks by unplanned CS was also analysed, 30.0% of whom were delivered under GA. These antenataly compromised foetuses had increased risks of both intubation (RR = 3.4, 95% CI 2.4, 4.8) and of Apgar5 <7 (RR = 4.3, 95% CI 3.1, 5.9), when the CS was performed under GA.

GA was more frequent in other public hospitals for all three of these risk groups. For the low-risk repeat CS deliveries, 22.5% were performed under GA in other public hospitals compared with 14.4% in large public hospitals and 9.0% in private hospitals. For the 'failure to progress' risk group, the rate of GA was 25.4% in other public hospitals, 9.6% in large public hospitals and 9.4% in private hospitals. For unplanned CS for foetal distress, 39.0% of deliveries were by GA at other public hospitals, 24.0% of deliveries at large public hospitals, and 14.9% of deliveries in private hospitals.

**Figure 1:**
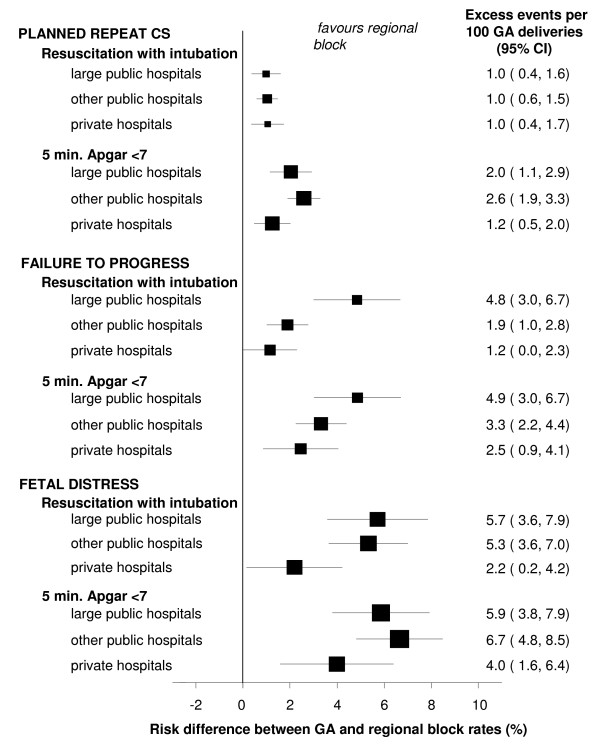
**Risk differences for neonatal outcomes, for caesarean section under general anaesthesia compared with regional block, by caesarean section indication and hospital level**.

Figure 1 shows the risk differences for resuscitation with intubation and for an Apgar5 <7 for each CS indication group, by hospital category. For all of the CS indications and across all of the hospital levels, the results favoured regional block over GA. The planned repeat CS group showed no variation by hospital level in difference in intubation rates (heterogeneity I^2 ^= 0%), but there was strong heterogeneity for the Apgar5 score (I^2 ^= 75%). This was influenced by private hospitals, which had both the lowest rate of Apgar5 <7 scores after GA (1.4%) and the highest rate after regional block (0.2%), resulting in the smallest risk difference (1.2 extra Apgar5 <7 scores per 100 deliveries under GA). For the failure to progress group, there was strong heterogeneity in the risk differences for intubation (I^2 ^= 82%) and the Apgar5 outcome (I^2 ^= 52%). The heterogeneity was driven by the relatively high rate of intubation (5.4%) and Apgar5 <7 (5.4%) in large public hospitals after GA, whereas other public and private hospitals had intubation rates of <2.5% after GA. For the foetal distress indication group, there was strong heterogeneity in the risk differences for intubation (I^2 ^= 72%) and weak heterogeneity for the Apgar5 outcome (I^2 ^= 7%). This was mainly due to the relatively low rates of intubation (3.9%) and Apgar5 <7 (5.7%) in private hospitals after GA for this group.

## Discussion

This is the largest study to compare the effect of anaesthesia methods for CS on neonatal outcome, and controls for confounding by specification of both pregnancy risk and indication for CS. We have shown that there are significant risks to the neonate of both resuscitation requiring intubation and of a poor Apgar score at 5 minutes, for a range of delivery indications. The greatest RR of both adverse outcomes occurred in the low-risk planned repeat CS deliveries under GA, but the greatest excess in risk attributable to GA was for emergency deliveries for foetal distress where the infant would already have been compromised to some extent. Not only did GA increase the risk of intubation, but it also increased the probability that an intubated infant's Apgar5 score would be <7 compared with an infant intubated after a regional block.

Similarly to reports from other developed countries [7,12,13], the use of GA for CS in NSW has decreased and the use of spinal anaesthesia has become more widespread. However, GA was still used for 12.6% of CS deliveries in 2006 across all levels of hospitals in the state. This study provides strong evidence that the guidelines recommending regional block over GA for most CS are prudent and beneficial for neonates as well as for mothers [1,2]. The RR of both intubation and a low 5-minute Apgar score were greatly decreased if a regional block was used for all three of the defined risk groups and across all three hospital levels. While this study is based on observational data, it did include a large number of deliveries drawn from an entire population. The CS indication groups make the comparisons meaningful and control for confounding. The result for the high-risk foetal distress group is arguably still subject to confounding by indication since the precise cause and degree of the foetal distress is not reported, although haemorrhage and maternal hypertensive disorders and preterm deliveries were excluded. The relative risk of an Apgar5 <7 strongly favoured regional block for this group (RR = 4.7 if GA was used), and it seems unlikely that selection bias could explain this away. The increase in rates of intubation as the urgency of the indication for CS increased was consistent with the increase in rates of Apgar5 <7.

Previous population-based studies of anaesthesia for delivery in Tasmania did find significantly increased risks of intubation and Apgar1 <4 for both repeat and primary CS under GA [14], and an RR of intubation = 10.8 (95% CI 3.2, 36.0) when emergency CS was performed under GA [15]. A recent US study, which included births from 14 university-based hospitals, showed an increased odds of both Apgar1 ≤ 3 and Apgar5 ≤ 3 but did not specify the indications for CS [3]. Other observational studies have found an increased need for resuscitation when GA is used [4]. However, these had limited statistical power for an Apgar5 outcome, and this continued to be a limitation in two more recent studies [16,17]. Controlling or stratifying for the indication for CS is also usually absent. A study of 3940 deliveries in a tertiary referral hospital did use three CS indication groups (that is, elective, urgent and emergency), which approximated the categories in this study. That study found that there was a significant increase in rates of intubation and low Apgar5 score for urgent and emergency CS, but was under-powered for the elective deliveries, and did not control for factors such as gestational age [18]. Randomised trials of anaesthesia in CS not only have had small numbers of deliveries [5], but may also have limited generalisability [19]. For instance, the only randomised trial with more than five Apgar5 <7 events was of pregnancies affected by severe pre-eclampsia [20].

A limitation to this study is that infant records were only available up to separation from the birth hospital, so longer term outcome data was not available. An Apgar5 <7 is usually associated with birth asphyxia [21], but it is unclear whether an Apgar5 <7 affected by GA has the same prognostic value. That the setback due to GA could be temporary is plausible to some extent for low-risk infants. The greatest burden may be on those infants already compromised *in utero*, as indicated by foetal distress, who in this study had significantly increased risks of both intubation and a low Apgar5 score if the delivery was performed under GA.

A strength of this study is the large, well validated, population-based data. Like all such databases, there could be some under-reporting of risk factors for CS. However, for maternal hypertension it has been shown that the more severe manifestations, which result in maternal morbidity, are more likely to be reported [22]. If this holds true generally for pregnancy complications, risk factors are likely to have been well reported in cases of adverse neonatal outcomes. Variations in experience and skill level of anaesthetists or obstetricians may have been partly responsible for differences in the risks associated with GA, as evidenced by the heterogeneity of outcomes by hospital category. However, all comparisons across all hospital levels favoured the use of regional block over GA.

## Conclusion

Concerns about the effects of GA on the neonate have mostly focused on acid-base status, resuscitation and the Apgar score at 1 minute, with the presumption that the effect of GA on the infant is short lived [6]. The increased rates of neonatal intubation after GA in this study represent harm in and of itself, and the persistence of low 5-minute Apgar scores suggests that deleterious effects may last longer than the immediate aftermath of delivery. The greatest absolute increase in the rate of intubation and of a 5-minute Apgar score <7 for deliveries performed under GA occurred in the most vulnerable infants: those that were delivered by emergency CS because of foetal distress. Clinicians considering the use of GA for a CS delivery should be aware of these possible consequences for the infant, for both planned and emergency CS.

## Abbreviations

CI: confidence interval; CS: caesarean sections; GA: general anaesthesia; MDC: Midwives Data Collection; NSW: New South Wales; RR: relative risk

## Competing interests

The authors declare that they have no competing interests.

## Authors' contributions

CSA planned the study, performed the analyses, and wrote the manuscript. JRB, WBG, GEK and CLR planned the analyses and edited the manuscript. SLL researched the study and edited the manuscript.

## Pre-publication history

The pre-publication history for this paper can be accessed here:


